# Mass spectrometry-based proteomics delivers in-depth proteome profiling of FFPE lung cancer biopsies from single glass slides

**DOI:** 10.1038/s41698-026-01517-8

**Published:** 2026-05-29

**Authors:** Olena Berkovska, Igor Schliemann, Georgios Mermelekas, Nazlı Ezgi Özkan, Mahnaz Nikpour, Vilde Drageset Haakensen, Åslaug Helland, Janne Lehtiö, Lukas M. Orre

**Affiliations:** 1 Department of Oncology and Pathology, Karolinska Institutet, SciLifeLab, Solna, Sweden; 2 Department of Pathology and Cancer Diagnostics, Karolinska University Hospital, Stockholm, Sweden; 3 Department of Oncology, Oslo University Hospital, Oslo, Norway

**Keywords:** Biological techniques, Biomarkers, Cancer, Computational biology and bioinformatics, Oncology

## Abstract

Clinical proteomics has the potential to add a valuable data layer to genomic and histopathological analyses in precision oncology, but its application to limited clinical material remains challenging. Here, we demonstrate that state-of-the-art mass spectrometry-based proteomics enables in-depth proteomic profiling of formalin-fixed paraffin-embedded (FFPE) clinical samples, including small diagnostic biopsies and single tissue sections mounted on glass slides. Despite minimal input material, single-slide analyses of clinical lung tumor specimens yielded biologically and clinically informative data, supporting detection of actionable proteins, immune-related signatures, and multivariate biomarkers. These results establish the feasibility of proteomics for retrospective FFPE studies and routine clinical practice, expanding opportunities for biomarker discovery and precision medicine from scarce tissue material.

Proteomics has emerged as a promising complement to immunohistochemistry (IHC) and genomic profiling in cancer research and diagnostics, offering deeper insight into tumor biology and therapeutic response^1–6^. To date, most proteomics studies have relied on fresh-frozen tissue; however, formalin-fixed paraffin-embedded (FFPE) samples are the most widely used material in clinical practice, with millions of specimens stored in biobanks. FFPE material therefore holds great potential for the large-scale studies needed to decipher cancer complexity and advance precision medicine. Substantial progress has been made in mass spectrometry (MS)-based proteomics of FFPE samples, driven by advances in instrument sensitivity and improved workflows^7–10^. Nonetheless, performance in clinical settings with limited material, such as biopsies and/or tissue sections mounted on glass slides, remains unclear.

In this study, we analyzed a cohort of FFPE tumor biopsy specimens from unresectable lung cancer using a single 4-μm section on a glass slide per patient (“single-slide cohort”, *n* = 68; Fig. 1A and Supplementary Table 1). An additional, smaller cohort comprising multiple FFPE sections from biopsies or surgically resected tumor specimens served as a reference (“multi-section cohort”, *n* = 15). The samples were analyzed using label-free data-independent acquisition (DIA) proteomics on two state-of-the-art MS instruments (Orbitrap Astral and timsTOF HT). In addition, multi-section cohort samples were analyzed on an older-generation MS instrument (Orbitrap Exploris).

**Fig. 1:**
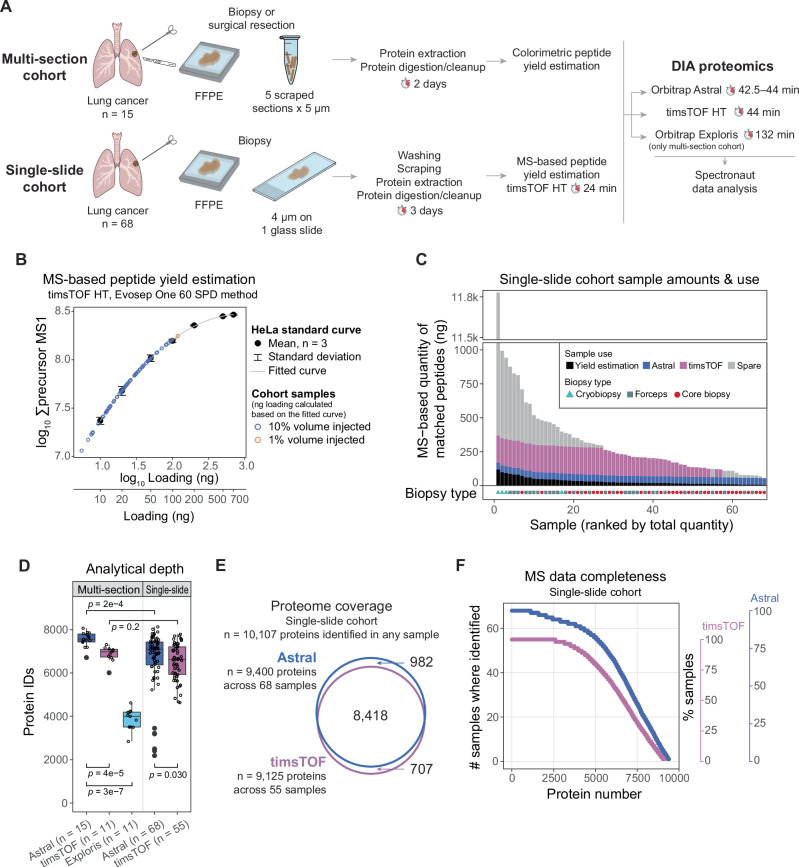
MS-based analysis of formalin-fixed, paraffin embedded (FFPE) clinical tumor samples delivers in-depth, proteome-wide profiling. **A** Schematic overview of the study design. **B** HeLa-based standard curve relating injected peptide amount to total MS1 signal, with single-slide cohort samples (*n* = 68) projected onto the curve to estimate injected peptide amounts. The curve was modeled using a quadratic polynomial regression relating log_10_-transformed values. **C** Estimated peptide yields extracted from glass slides in the single-slide cohort (calculated as shown in **B**) and the proportion of each sample used for the different analyses. **D** Number of proteins (gene-centric) identified per sample in MS-based analyses of the multi-section and single-slide cohorts. *P* values were calculated using two-sided Wilcoxon rank-sum test. The number of samples in each dataset is indicated in the figure. **E** Total number of proteins (gene-centric) identified in the single-slide cohort analyses and the overlap between the Astral and timsTOF datasets. **F** Number of samples in which each protein was identified in the single-slide cohort analyses.

Sample preparation for both cohorts followed the same general workflow, with two key differences: inclusion of a wash step and the use of an MS-based peptide amount estimation instead of a colorimetric assay for the single-slide cohort (Fig. 1A). In a small pilot analysis (n = 6) of the single-slide cohort, we observed substantially higher MS signal from skin keratins compared with the multi-section cohort (Supplementary Fig. 1A, B). High-abundance proteins present a challenge in MS proteomics due to ion suppression of lower-abundance peptide signals. We hypothesized that the elevated skin keratin signal resulted from contamination accumulated during handling and long-term storage of glass slides. Accordingly, we introduced a glass-washing step using a mild non-ionic detergent for preparation of the full cohort. Although this step modestly reduced skin keratin levels, the difference was not significant, and the proportion of MS signal originating from a small number of high-abundance proteins remained significantly higher than in the multi-section cohort (Supplementary Fig. 1B). Further protocol optimization may improve these results, but was beyond the scope of this study due to limited material availability. Therefore, we will consider skin keratin contamination as a quality control parameter in the downstream analysis.

The second protocol adaptation for the single-slide cohort involved peptide yield estimation. Colorimetric assays are commonly used in bulk proteomics to quantify total peptide yield and normalize injection amounts; however, they require relatively large amounts of material (several hundred nanograms), and their accuracy can be compromised by interference from non-peptide components in FFPE samples. To address these limitations, we employed an MS-based estimation strategy similar to that described by Tüshaus et al.^7^. Briefly, single-slide cohort samples were analyzed using a fixed injection volume alongside HeLa standards (10–700 ng injections). A HeLa standard curve was generated using summed MS1 precursor signal, and peptide amounts in clinical samples were calculated from this curve (Fig. 1B). We refer to these values as “MS-based quantity of matched peptides”, as this estimate does not account for fragmented, cross-linked, or otherwise non-canonical peptides.

MS-based matched peptide yields in the single-slide cohort ranged from 55 ng to 1 μg per sample, except for one large cryobiopsy yielding more than 10 μg (Fig. 1C). These values were substantially lower than those obtained from the multi-section cohort, in which 5–250 μg of colorimetrically quantified peptides were extracted (Supplementary Fig. 1C). Notably, biopsy size showed a moderate-to-strong correlation with the estimated peptide yield (Supplementary Fig. 1D). Although biopsy length cannot be used to reliably normalize injection amounts for full MS analyses, these correlations can inform pre-run peptide yield estimation to ensure the samples fall within the HeLa standard curve. Importantly, using only 10% of each sample for the initial yield estimation left sufficient material for at least one full MS analysis of all single-slide cohort samples (Fig. 1C). The selection of injection amounts for full MS analyses balances a trade-off between analytical depth and long-term instrument performance, as well as the number of samples with sufficient material for equal injections: higher peptide loading can increase protein identifications but also necessitate more frequent instrument maintenance. Based on these considerations, we selected a 50-ng injection for the Astral instrument. For the timsTOF analysis, we used 200 ng when sufficient material was available; otherwise, we used the remaining sample amount, but no less than 50 ng.

Across the full single-slide cohort analyses, we obtained deep proteomic coverage, identifying up to approximately 8000 proteins (gene-centric) per sample and 10,000 proteins across the cohort (Fig. 1D–F). The median number of proteins was 7097 and 6629 in the Astral and timsTOF datasets, respectively. While the analytical depth in the single-slide cohort was significantly lower than in the multi-section cohort (median 7097 vs 7557 and 6629 vs 6980 proteins in the Astral and timsTOF datasets, respectively; Fig. 1D and Supplementary Fig. 1E), higher injection amounts, as expected, increased the number of identifications (Supplementary Fig. 1F, G). This analytical depth reflected a compromise aimed at maximizing the cohort size by minimizing the amount of injected material and using all available (unselected) samples. For comparison, another study optimized for maximal analytical depth in rapid DIA analyses of FFPE tissues reported a median of approximately 9100 identified proteins when injecting 1 μg of peptides from a small cohort of 13 lung tumors^8^.

We next evaluated the effect of skin keratin contamination on analytical depth and quantification validity. Skin keratin levels showed a strong negative correlation with the number of identified proteins, an expected effect for high-abundance proteins (Supplementary Fig. 2A). Consistently, this effect was even more pronounced in a broader analysis of the 20 most abundant proteins (Supplementary Fig. 2A). In our cohort, HBB (hemoglobin subunit beta) was a notable contributor to MS signal, highlighting the well-known challenge of plasma and blood contamination in MS samples (Supplementary Fig. 2B). Nonetheless, despite the lack of preselection for MS analysis, over 90% of samples yielded more than 5,000 identified proteins, with only six of 68 samples classified as outliers in analytical depth.

To exemplify quantitative validity and assess the impact of high abundance proteins on quantification, we evaluated concordance among six proteins in the MCM complex, whose abundances are expected to correlate^1^. Across the cohort, quantitative agreement was strong, with low overall variability among MCM complex members (Supplementary Fig. 2C, D). However, standard deviation between MCM proteins tended to increase in samples with higher levels of skin keratins. This suggests that caution should be exercised when interpreting quantitative data from low quality samples such as those contaminated with blood, plasma, or skin keratins.

We next evaluated the biological and clinical relevance of the generated proteomic profiles. To provide biological context, we integrated publicly available protein lists of interest and clinical annotations with our MS data (Figs. 2A, E and 3D). MS-based quantification of histological markers routinely used in pathology to distinguish adenocarcinoma (AC) from squamous cell carcinoma (SCC) enabled resolution of AC and SCC samples, with strong concordance between the Astral and timsTOF datasets (Fig. 2B, C and Supplementary Fig. 2E). While previous studies have reported disagreement between proteomics and IHC-based histology annotations for outlier cases^5^, the separation of the two histological groups in the single-slide cohort was nonetheless not as complete as expected. To investigate this further, we performed a subgroup analysis based on the biopsy type and observed a stronger distinction between AC and SCC samples in core biopsies than forceps biopsies (Fig. 2C). Forceps biopsies can pinch through the bronchial epithelium, introducing normal tissue into samples and thus dilute the tumor signal. This raises a concern regarding the informativeness of bulk proteomics of biopsy samples without corresponding H&E or IHC evaluations. Macro- or microdissection of the samples could reduce the signal contamination from adjacent normal tissue and enrich for tumor cells. Furthermore, the availability of the corresponding scanned images could help inform the proteomics data analysis regarding immune and stromal infiltration of the tumor, thus facilitating filtering or deconvoluting the data to reduce dilution of the tumor signal.

**Fig. 2:**
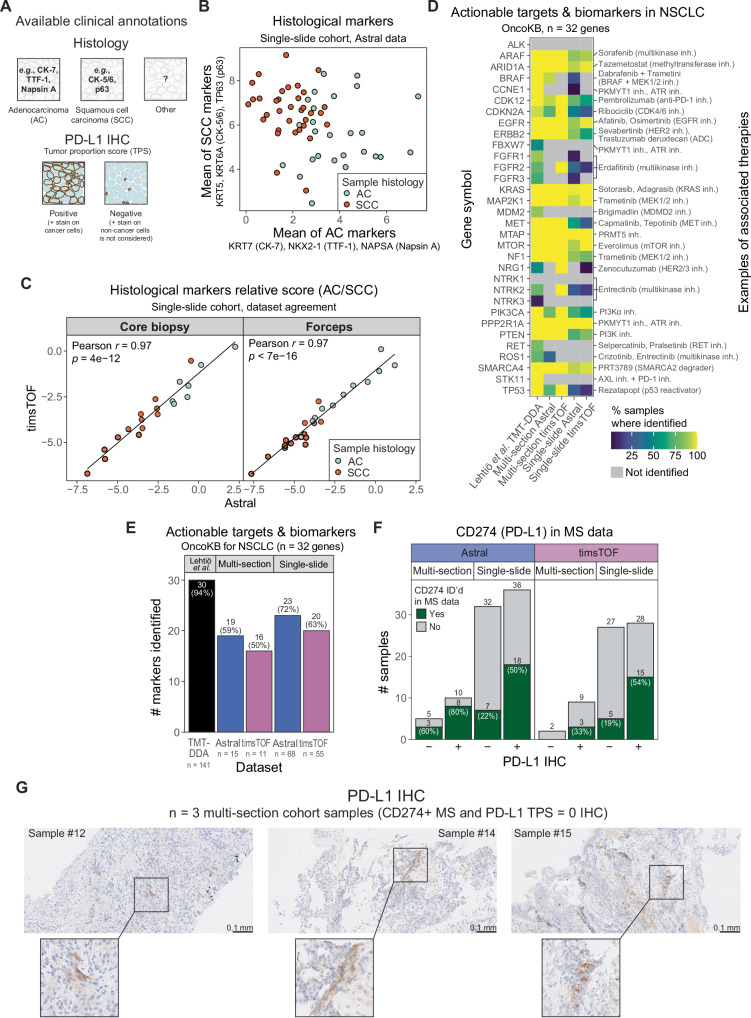
MS-based analysis of single FFPE slides provides biologically sound data and identifies clinically actionable targets. **A** Schematic overview of the available clinical annotations used to provide context for the proteomic observations. **B** MS-based quantitative signature of histological markers in adenocarcinoma (AC, *n* = 26) and squamous cell carcinoma (SCC, *n* = 34) samples in the single-slide cohort Astral dataset. **C** Relative levels of histological markers (log-transformed adenocarcinoma marker score minus log-transformed squamous cell marker score, as shown in **B**) in core biopsies (left; n = 20) and forceps biopsies (right; *n* = 25) from the single-slide cohort in the Astral and timsTOF datasets. Identification of actionable drug targets and predictive biomarkers in non-small-cell lung cancer (NSCLC) as listed in the OncoKB database with **D** annotation of the sample percentage where identified and **E** summary statistics. An in-depth TMT-DDA dataset of 141 NSCLC tumors covering 13,975 proteins (Lehtiö et al.^5^) was used as a reference proteomics dataset. **F** Identification of CD274 (PD-L1) in MS proteomics data stratified by PD-L1 status, as determined by tumor proportion score (TPS) assessment performed in clinical routine, where TPS > 0 is positive (+) and TPS = 0 is negative (–) PD-L1 expression. **G** Representative PD-L1 immunohistochemistry (IHC) images from three multi-section cohort samples with tumor proportion score (TPS) = 0, but CD274 identified in MS proteomics data. PD-L1 staining is absent in tumor cells; positive staining is observed in macrophages and other stromal cells.

**Fig. 3:**
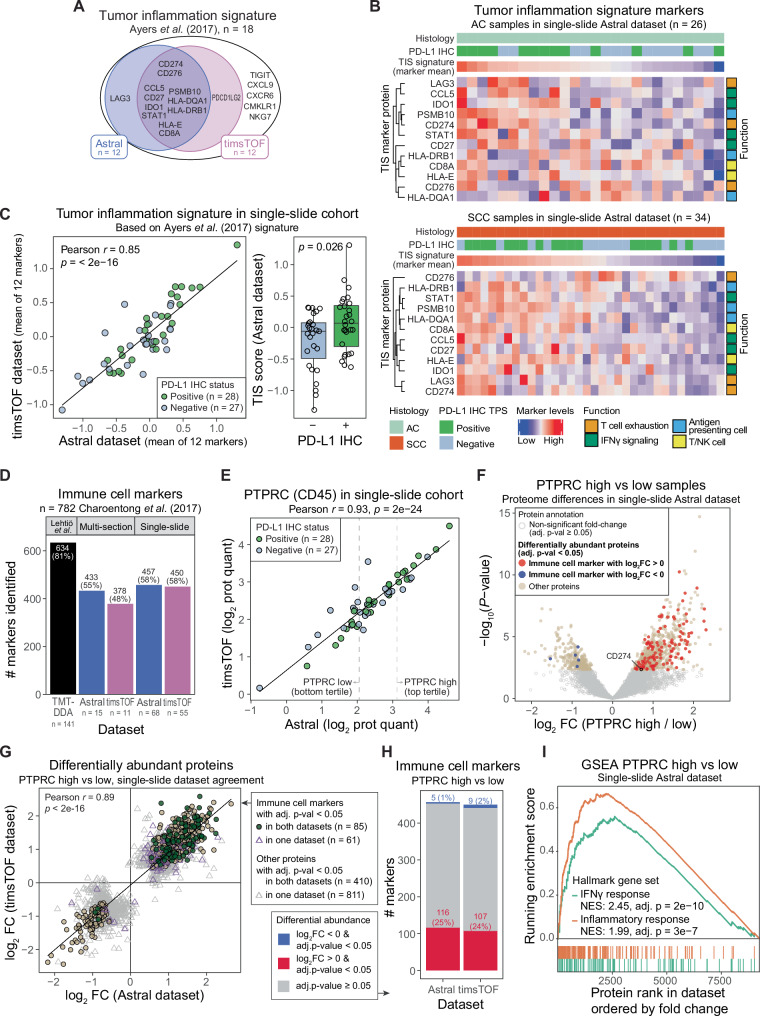
MS-based proteomics of single FFPE slides quantifies molecular signatures and provides opportunities for in-depth exploratory analyses. **A** Markers from the tumor inflammation signature (TIS) proposed by Ayers et al.^13^ and identified in the single-slide cohort. **B** Scaled levels of TIS proteins in adenocarcinoma (AC, top) and squamous cell carcinoma (SCC, bottom) samples in the single-slide Astral dataset. Columns ordered by the signature marker mean; rows clustered by hierarchical clustering using Spearman correlation distance. PD-L1 immunohistochemistry (IHC) annotation like in Fig. 2. **C** Mean of scaled TIS protein levels in single-slide Astral and timsTOF datasets (left) and stratified by PD-L1 status (right). *P* value was calculated using two-sided Welch two-sample *t*-test. **D** Identification of immune cell markers published by Charoentong et al.^17^. The same Lehtiö et al.^5^ reference dataset was used as in Fig. 2. **E** Protein quantities of an immune cell marker PTPRC (CD45) in the single-slide cohort. Tertiles were defined based on the Astral dataset and included samples not analyzed in the timsTOF dataset. **F** Differential abundance analysis using DEqMS comparing PTPRC high versus low tertiles in the single-slide cohort Astral dataset (*n* = 23 per group). Immune cell marker labels refer to the Charoentong et al.^17^ list. **G** Comparison of differentially abundant proteins like in **F** indicated in the Astral and/or timsTOF datasets (sample groups defined using Astral data). **H** Percentage of immune cell markers (Charoentong et al.^17^ list) that are differentially abundant in the PTPRC high versus low comparison in the Astral and/or timsTOF datasets. **I** Gene-set enrichment analysis (GSEA) using ranked fold changes in PTPRC high versus low tertile comparison in the single-slide cohort Astral dataset (shown in **F**), assessing enrichment of MSigDB interferon gamma (IFNγ) and inflammatory response hallmark gene sets^18^. FC fold change, NES normalized enrichment score.

We next assessed identification of clinically actionable drug targets and predictive biomarkers listed in the OncoKB database for lung cancer^11^. Across datasets, 63–72% of these markers were identified in the single-slide analyses, compared with 50–59% in the multi-section datasets, a difference most likely attributable to the small cohort size in the context of cancer heterogeneity (Fig. 2D, E). We then examined MS-based identification of PD-L1 in relation to tumor PD-L1 status determined by immunohistochemistry (IHC), which is routinely used to guide immunotherapy selection in lung cancer. CD274 (PD-L1) was identified in 80% of IHC-positive cases in the multi-section Astral dataset, but in only 33–54% of positive samples in the remaining datasets (Fig. 2F), suggesting limited sensitivity for CD274 detection in FFPE samples using the current workflows. Overall, CD274 was detected in 25–75% of samples across datasets. Notably, most identifications occurred in PD-L1 IHC-positive tumors (Fig. 2F), whereas the remaining detections likely reflect PD-L1 expression by non-cancer cells in the tumor microenvironment (which is not captured by the IHC scoring, Fig. 2G), or tumor heterogeneity, as the PD-L1 IHC staining was performed on tissue sections distinct from those used for MS analysis.

Individual biomarkers such as PD-L1 can inform clinical decision making; however, multivariate biomarkers are increasingly recognized as required to capture the complexity of tumor biology, therapy response, and resistance mechanisms for more precise therapy selection^12^. The ability to generate data at scale for quantitative analysis of marker sets and molecular signatures is a key strength of MS-based proteomics. Here, as a proof-of-concept, we demonstrate signature-level analyses using the tumor inflammation signature (TIS) composed of 18 markers proposed by Ayers et al.^13^, which characterizes the tumor immune microenvironment (TIME), a key determinant of response to immunotherapy (Fig. 3A, B)^14^. Although originally defined at the mRNA-level data and now implemented in RNA-based gene expression assays; proteomics offers several advantages over transcriptomics. Proteome-level information reflects the final molecular tumor phenotype and drug target levels more accurately due to frequently low mRNA-protein correlations^5,15^. Moreover, proteins are more stable than RNA, enabling more robust profiling of archived material^16^. In the single-slide cohort, the TIS score showed strong agreement between the Astral and timsTOF datasets (Fig. 3C). Notably, although the TIS scores were significantly higher in PD-L1-positive tumors than in PD-L1-negative, the difference was small, and the large variability in both groups highlights that TIME assessment provides information orthogonal to PD-L1 status, in agreement with what has been previously shown^5^. However, the ability to interpret the derived TIS scores is limited by the absence of tumor cellularity and histopathological assessment information.

Beyond molecular signature quantification, in-depth proteomics enables exploratory analyses; therefore, we next examined immune-related signals at a broader scale. Using immune cell-type gene sets published by Charoentong et al.^17^, including 782 proteins as a reference, we detected 58% of the markers in the single-slide cohort (Fig. 3D). We then stratified the single-slide cohort by abundance of the pan-immune cell marker PTPRC (CD45; Fig. 3E) and compared proteomic profiles between the top and bottom tertiles (Fig. 3F–H). In the Astral dataset, 25% of identified immune cell markers were significantly elevated in the PTPRC-high group, whereas only 1% were significantly lower. Finally, gene-set enrichment analysis (GSEA) using immune-related MSigDB hallmark gene sets^18^, as expected, revealed significant enrichment of IFNγ and inflammatory response pathways in the PTPRC-high group (Fig. 2I).

In conclusion, our study demonstrates that state-of-the-art MS instrumentation and optimized workflows enable rapid, in-depth proteomic analysis of clinical FFPE specimens, including small biopsies and limited material on single glass slides. Although multiple sections remain advantageous for maximizing analytical depth, single-slide analyses can still provide biologically and clinically informative data suitable for both targeted and exploratory applications.

We identified contamination from adjacent normal tissue and/or high abundance proteins to be key factors that determine sample quality in FFPE cancer biopsies on glass slides. These challenges may be mitigated through macrodissection and the use of freshly cut sections, respectively. Alternatively, laser capture microdissection (LCM) allows for more focused research questions through the selective analysis of tumor cells or other specific cell types^10,19,20^. Regions of interest can be selected either through manual annotation by a clinical pathologist, which limits throughput, or through automated image analysis, which requires expensive specialized equipment. While these techniques represent exciting developments in proteomics and enable unprecedented spatial resolution, they offer lower throughput and may be less compatible with clinical routines or large cohort studies. Nevertheless, bulk proteomics can be further strengthened by integrating complementary imaging and cell composition data. Based on our observations, we recommend careful handling of slides and FFPE sections to minimize contamination from skin and dust and, if possible, use macrodissection to enrich tumor regions. Furthermore, histological assessment of adjacent sections is crucial to support more accurate interpretation of proteomics data.

By demonstrating the quantification of clinically actionable proteins, immune-related markers, and multivariate signatures, our findings highlight the value of proteomics for capturing functional tumor phenotypes beyond single-marker assessments, consistent with growing evidence that complex biomarkers are essential for precision oncology. Importantly, we show that these capabilities extend to archived FFPE biopsies derived from unresectable cases, enabling retrospective analyses of clinically annotated cohorts despite limited tissue availability. This opens opportunities for detailed proteome analysis of previously underutilized specimen collections of biopsies from late-stage patient cohorts, a patient population where new oncology drugs are typically initially tested. Our study demonstrated that clinical proteomics is a practical and powerful modality for precision medicine and cancer research.

## Methods

### Clinical sample collection

The study was performed in accordance with the Declaration of Helsinki. The multi-section FFPE cohort comprised of 15 samples collected from lung cancer patients at the Karolinska University Hospital in Solna, Sweden. The surgical samples originated from resected lung tumors during routine treatment of patients. The biopsy samples (core needle or forceps) were collected during diagnostic procedures for suspected lung cancer patients. The study was approved by the Regional Ethical Review Board in Uppsala, Sweden (registration nos. 2021-01931 and 2024-04958-02). Informed consent was obtained from all the patients. The FFPE sample preservation was performed as part of the clinical routine. The samples were used for research only after routine diagnostic procedures were performed and sufficient material remained. Five consecutive sections (5 μm each) were then taken for and shortly before proteomic analysis. FFPE blocks were sectioned, and the sections were mounted on glass slides. Macrodissection was then performed to minimize non-tumor content by scraping off areas identified as non-tumor region by a qualified pathologist. The remaining parts of the sections were then scraped into Eppendorf tubes. All samples were processed rapidly, and the glass slides were not stored prior to analysis. PD-L1 staining was performed as part of clinical routine using SP263 (Ventana) antibody.

The single-slide FFPE cohort comprised of 68 samples collected from lung cancer patients at the Oslo University Hospital in Oslo, Norway. The biopsy samples (core needle, forceps, or cryobiopsy) were collected as part of the DART trial approved by the Regional Ethical Committee for Medical Health Research Ethics, REK South-East in Oslo, Norway (reference no. 48655). Informed consent was obtained from all the patients. The FFPE sample preservation was performed as part of the clinical routine with 4-μm tissue sections mounted on glass slides. The glass slides were initially prepared for other analyses, leftover slides were stored for several years, after which they were used for proteomic analyses.

The meta data for the samples, including histological and PD-L1 status annotations, peptide yields, generated datasets, and the number of identified proteins, is provided in Supplementary Data 1.

### Sample preparation

For the single-slide cohort, the slides were pre-washed with 0.01% n-Dodecyl-B-D-Maltoside (DDM) by dipping and shaking the slide in the solution for approximately 10 s, rinsing in water twice, and drying. The FFPE material was then scraped off using a sterile scalpel into Eppendorf tubes.

All samples were prepared for proteomics analysis using the FenoPrep^TM^ kit (Cat# 532001, FenoMark Diagnostics AB, Sweden) per the manufacturer’s instructions. The method utilized xylene-free deparaffinization based on high-temperature, high-energy ultrasonication^21^. Briefly, the lysis buffer was added to the samples, heated under shaking (95 °C, 400 rpm, 30 min), after which the sample tubes were placed in the VialTweeter Ultrasonic processor (Hielscher Ultrasonics GmbH, Germany; settings: amplitude 100%, pulsation mode 100%, 10 cycles of 60 s on/30 s off). The samples were then heated under shaking (95 °C, 400 rpm, 30 min). The samples were then placed in the ultrasonic processor for 5 more cycles. The samples were centrifuged (13,000 × *g*, 10 min) and the supernatant was transferred for further processing. The proteins were alkylated. Thereafter, on-bead digestion using Lys-C and trypsin and clean-up using magnetic beads were performed. For the multi-section cohort, the peptide concentration was measured using a microBCA assay with HeLa digest as the standard. For the single-slide cohort, MS-based peptide yield estimation was performed (see next section).

### MS-based peptide yield estimation using timsTOF HT

A timsTOF HT mass spectrometer (Bruker) connected to an EVOSEP LC system via the CaptiveSpray 2 source was used for DIA-PASEF LC-MS/MS analysis. Each sample was analyzed after transfer of 10% peptide solution volume on Evotips following the manufacturer´s instructions (except for one sample where 1% of the volume was used, loading amounts are provided in the supplementary materials). The peptides were separated on a PepSep C18 column (Bruker, 8 cm × 150 µm, 1.5 µm), using the 60SPD fixed method. For the dia-PASEF analysis the window scheme was calculated using the py_diAID tool (https://github.com/MannLabs/pydiaid). The capillary voltage was set at 1500 V, stepping collision energy at 32, 40, 50 eV, dia-PASEF scan range 100–1700 *m*/*z* in positive mode, and IMS service ramp time of 100 ms.

### DIA analysis using Orbitrap Astral

For the multi-section cohort, an Orbitrap Astral coupled to a Vanquish NEO System (Thermo Fisher Scientific) was used. The injection volume was 2 μl for each sample containing 500 ng of peptides. The samples were trapped on a C18 guard-desalting column (Acclaim PepMap NEO, 300 μm × 5 mm, nanoViper, C18, 5 µm, 100 Å) and separated on a 25 cm long C18 column (25 cm Aurora Ultimate XT column, C18, 1.7 μm, 75 μm × 25 cm). The nanocapillary solvent A was 0.1% formic acid in water; and solvent B was 0.1% formic acid in acetonitrile. At a constant flow of 0.25 μl/min, the curved gradient went from 6% B up to 40% B in 32 min, followed by a steep increase to 100% B in 5 min.

For the single-slide cohort, an Orbitrap Astral System (Thermo Fisher Scientific) coupled to an EVOSEP LC system was used. Each sample was analyzed after the transfer of 50 ng of peptides on Evotips following the manufacturer´s instructions. The peptides were separated on a PepSep C18 column (Bruker, 15 cm × 75 µm, 1.5 µm), using the 30SPD fixed method with a total method duration of 44 min.

The standard MS parameters for DIA acquisition were set as follows: a spray voltage of 2000 V, no sheath or auxiliary gas flow, and a heated capillary maintained at 280 °C. Full-scan mass spectra were acquired with a scan range of 380–980 *m*/*z*, an AGC target value of 300%, maximum injection time (IT) of 10 ms, and a resolution of 240,000. The DIA windows were set to 2 *m*/*z* in a precursor mass range of 380–980 *m*/*z* and subjected to fragmentation (nCE: 25%) with a scan range of 145–1450 *m*/*z*, maximum IT of 3 ms, AGC target value of 500%.

### DIA analysis using timsTOF HT

A timsTOF HT mass spectrometer (Bruker) connected to an EVOSEP LC system via the CaptiveSpray 2 source was used for DIA-PASEF LC-MS/MS analysis.

For the multi-section cohort, 11 samples were analyzed after transfer of 500 ng of peptides on Evotips following the manufacturer´s instructions. The peptides were separated on a C18 IonOpticks column (particle size 1.6 µm, 75 µM µm ID, 15 cm length) at a flow rate of 0.25 µl/min; solvent A (0.1% formic acid in water), solvent B (0.1% formic acid in acetonitrile), using the 30SPD fixed method with a total method duration of 44 min.

For the single-slide cohort, 56 samples sample was analyzed after transfer of 50–200 ng of peptides on Evotips following the manufacturer´s instructions (loading amounts are provided in the Supplementary Data 1). The peptides were separated on a PepSep C18 column (Bruker, 15 cm × 75 µm, 1.5 µm), using the 30SPD fixed method with a total method duration of 44 min.

For the dia-PASEF analysis the window scheme was calculated using the py_diAID tool (https://github.com/MannLabs/pydiaid). The capillary voltage was set at 1500 V, stepping collision energy at 32, 40, 50 eV, dia-PASEF scan range 100–1700 *m*/*z* in positive mode, and IMS service ramp time of 100 ms.

One injection in the single-slide cohort failed, resulting in the final dataset of 55 samples.

### DIA analysis using Orbitrap Exploris 480

An Orbitrap Exploris 480 coupled to a Dionex UltiMate™ 3000 RSLCnano System was used System (Thermo Fisher Scientific). The injection volume was 2 µl for each sample containing 2 µl. Samples were trapped on a C18 guard-desalting column (Acclaim PepMap 100, 75 μm × 2 cm, nanoViper, C18, 5 µm, 100 Å) and separated on a 25-cm long C18 column (25 cm Aurora Ultimate XT column, C18, 1.7 μm, 75 μm × 25 cm). The nanocapillary solvent A was 0.1% formic acid in water; and solvent B was 0.1% formic acid in acetonitrile. At a constant flow of 0.25 μl/min, the curved gradient went from 6% B up to 40% B in 120 min followed by a steep increase to 100% B in 5 min. The standard MS parameters for DIA acquisition were set as follows: a spray voltage of 1900 V, no sheath or auxiliary gas flow, and a heated capillary maintained at 275 °C. Full-scan mass spectra were acquired with a scan range of 375–1175 *m*/*z*, an AGC target value of 100%, maximum injection time (IT) of 100 ms, and a resolution of 120,000. The DIA windows were set to 25 *m*/*z* in a precursor mass range of 375–1175 *m*/*z* and subjected to fragmentation (nCE: 28%) by HCD with a scan range of 145–1450 *m*/*z*, maximum IT of 100 ms, AGC target value of 100%, and a resolution of 15,000.

### DIA data search and preprocessing

DIA data were analyzed in Spectronaut (Biognosys, v.20) using directDIA mode and searched against ENSEMBL protein database (GRCh38.pep.all). The peptide quantities were summarized into proteins (gene-centric) using the MaxLFQ algorithm and only gene-specific peptides. The data was normalized using local normalization. Both imputed (global imputing) and non-imputed data were exported. Additionally, precursor-level non-imputed and non-normalized data, as well as the total ion chromatograms, were exported. Non-imputed data were used for identification analyses, while imputed exports were used as quantitative data. Total ion chromatograms and precursor-level exports were used for MS signal calculations; additionally, precursor-level data were used to assess the contribution of high-abundance proteins.

### Downstream bioinformatics analysis

All downstream bioinformatics analyses were performed in R (v. 4.5.2). To calculate the sample-wise normalized protein quantities, the MS2 area values exported from Spectronaut were divided by the median of all quantification in the respective sample; thereafter, the quantities were log_2_ transformed. For high abundance protein analysis, precursor-level MS1 data were exported. For the total ion chromatogram area under the curve (TIC AUC) analysis, the TIC chart data were exported from Spectronaut. Thereafter, the AUC was calculated using the AUC() function (trapezoid method) from the DescTools R package (v. 0.99.60). The differential abundance analysis was performed using the DEqMS package^22,23^ (v. 1.26.0) using imputed data and median precursor counts per protein. *P* values were adjusted using the BH method. The gene set enrichment analysis (GSEA) was performed using the GSEA() function from the clusterProfiler R package^24^ (v. 4.16.0) based on the ranked fold-change values derived from the DEqMS analysis. The hallmark gene sets originated from the MSigDB database^18^ (msigdbr R package v. 25.1.1). All the used statistical tests and the corresponding sample sizes are specified in the figures and figure legends.

### Public domain datasets

The Charoentong et al. immune cell markers were taken from the supplementary tables of the original publication^17^. The IFN gamma response and inflammatory response hallmark gene sets originated from the MSigDB^18^.

## Supplementary information

Supplementary materials

Supplementary Data 1

## Data Availability

MS proteomics data have been deposited on the ProteomeXchange Consortium via the PRIDE partner repository with the dataset identifiers PXD071948 (multi-section cohort) and PXD072014 (single-slide cohort).
