# Supplementary material for: Mass spectrometry-based proteomics delivers in-depth proteome profiling of FFPE lung cancer biopsies from single glass slides
Source: NPJ Precis Oncol. 2026 May 29;10:196. doi: 10.1038/s41698-026-01517-8 (PMC13221448; doi:10.1038/s41698-026-01517-8)
Supplement: Supplementary file 1 — Supplementary materials [file 41698_2026_1517_MOESM1_ESM.pdf]

Supplementary materials related to:

Mass spectrometry-based proteomics delivers in-depth proteome profiling of FFPE lung cancer biopsies from single glass slides

Authors and Affiliations

Olena Berkovska[1], Igor Schliemann[1,2], Georgios Mermelekas[1], Nazlı Ezgi Özkan[1], Mahnaz Nikpour[1], Vilde Drageset Haakensen[3], Åslaug Helland[3], Janne Lehtiö[1]✉, Lukas M. Orre[1]✉

[1] Department of Oncology and Pathology, Karolinska Institutet, SciLifeLab, Solna, Sweden

[2] Department of Pathology and Cancer Diagnostics, Karolinska University Hospital, Stockholm, Sweden

[3] Department of Oncology, Oslo University Hospital, Oslo, Norway

✉ e-mail: lukas.orre@ki.se, janne.lehtio@ki.se

List of Supplementary Information

- Supplementary Data 1 legend
- Supplementary Table 1
- Supplementary Figure 1
- Supplementary Figure 2

Supplementary Data 1 Spreadsheet file with meta data and key analytical information of multi-section and single-slide cohorts. Sheet “README”: Description of the remaining sheets, including clarification of the column names; **Sheet “MultiSection”**: sample information for the multi-section cohort; **Sheet “SingleSlide”**: sample information for the single-slide cohort.

Supplementary Table 1. Cohort characteristics.

	**Multi-section cohort (N = 15)**	**Single-slide cohort (N = 68)**
Median age, years (range)	75 (48–85)	68 (36–85)
Sex		
Male	7 (47%)	39 (57%)
Female	8 (53%)	29 (43%)
Histology		
Adenocarcinoma	15 (100%)	26 (38%)
Squamous cell carcinoma	0	34 (50%)
Other or NA	0	8 (12%)
Stage		
I	5 (33%)	0
II	3 (20%)	1 (1%)
III	3 (20%)	65 (96%)
IV	4 (27%)	0
Unknown	0	2 (3%)
PD-L1 status		
Negative	5 (33%)	32 (47%)
Positive	10 (67%)	36 (53%)
TPS 1–49	5 (33%)	*NA*
TPS ≥50	5 (33%)	*NA*
Sample type		
Surgical	7 (47%)	0
Biopsy	8 (53%)	68 (100%)
Core biopsy	5 (33%)	32 (47%)
Forceps biopsy	3 (20%)	32 (47%)
Cryobiopsy	0	4 (6%)
MS data		
Astral	15 (100%)	68 (100%)
timsTOF	11 (73%)	55 (81%)
Exploris	11 (73%)	*NA*

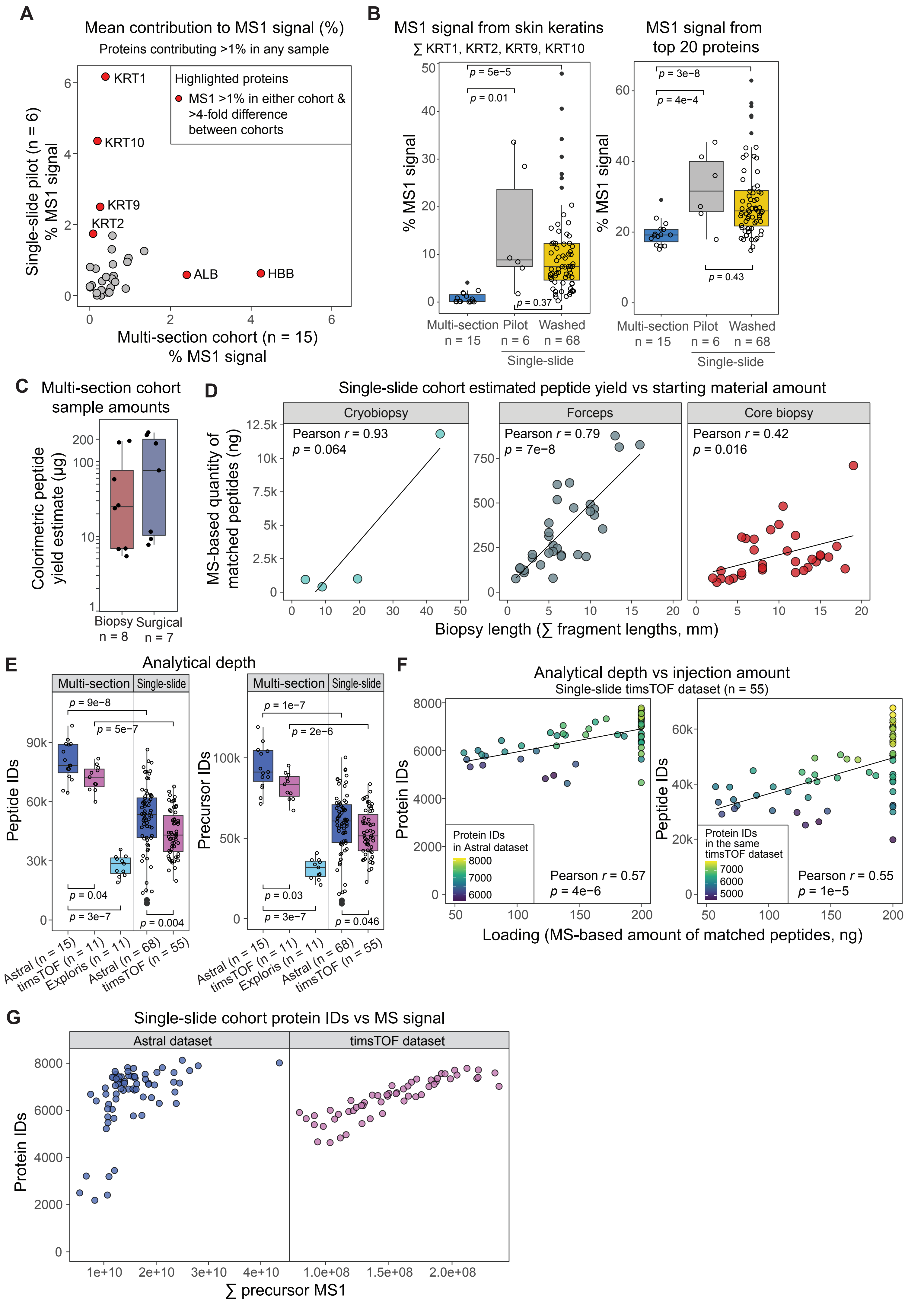
A
Mean contribution to MS1 signal (%)
Proteins contributing >1% in any sample
Highlighted proteins
MS1 >1% in either cohort & >4-fold difference between cohorts
KRT1
KRT10
KRT9
KRT2
ALB
HBB
Single-slide pilot (n = 6) % MS1 signal
Multi-section cohort (n = 15) % MS1 signal
B
MS1 signal from skin keratins
∑ KRT1, KRT2, KRT9, KRT10
MS1 signal from top 20 proteins
% MS1 signal
p = 5e−5
p = 0.01
p = 0.37
p = 3e−8
p = 4e−4
p = 0.43
Multi-section n = 15
Pilot n = 6
Washed n = 68
Single-slide
C
Multi-section cohort sample amounts
Colorimetric peptide yield estimate (µg)
Biopsy n = 8
Surgical n = 7
D
Single-slide cohort estimated peptide yield vs starting material amount
Cryobiopsy
Pearson r = 0.93
p = 0.064
Forceps
Pearson r = 0.79
p = 7e−8
Core biopsy
Pearson r = 0.42
p = 0.016
MS-based quantity of matched peptides (ng)
Biopsy length (∑ fragment lengths, mm)
E
Analytical depth
Multi-section
Single-slide
Peptide IDs
Precursor IDs
p = 9e−8
p = 5e−7
p = 0.04
p = 3e−7
p = 0.004
p = 1e−7
p = 2e−6
p = 0.03
p = 3e−7
p = 0.046
Astral (n = 15)
timsTOF (n = 11)
Exploris (n = 11)
Astral (n = 68)
timsTOF (n = 55)
F
Analytical depth vs injection amount
Single-slide timsTOF dataset (n = 55)
Protein IDs
Peptide IDs
Protein IDs in Astral dataset
Protein IDs in the same timsTOF dataset
Pearson r = 0.57
p = 4e−6
Pearson r = 0.55
p = 1e−5
Loading (MS-based amount of matched peptides, ng)
G
Single-slide cohort protein IDs vs MS signal
Astral dataset
timsTOF dataset
Protein IDs
∑ precursor MS1

Supplementary Figure 1. Analytical characteristics of the MS-based analysis of formalin-fixed, paraffin embedded (FFPE) clinical tumor samples (related to Figure 1). A) High abundance proteins (defined as contributing more than 1% of total MS1 signal in any sample, n = 31) and their mean contribution to the total MS1 signal across the multi-section and single-slide cohorts. **B)** Percentage of MS1 signal attributed to high-abundance proteins: four skin keratins (left) and top 20 proteins by signal (right) in the multi-section cohort, pilot analysis of the single-slide cohort, and the full analysis of the single-slide cohort, including a slide washing step. P values were calculated using Wilcoxon rank-sum test. The number of samples in each dataset is indicated in the figure. **C)** Peptide yield per sample in the multi-section cohort (n = 15) as determined using a colorimetric assay, grouped by sample type: biopsy (core or forceps, n = 8) and surgical material (n = 7). **D)** Peptide yields (as determined using the MS-based approach using HeLa standards) in the single-slide cohort in relation to the size of the biopsy on the slide, grouped by biopsy type: cryobiopsy (n = 4), forceps biopsy (n = 32) and core biopsy (n = 32). **E)** Number of peptides (left) and precursors (right) identified per sample in MS-based analyses of the multi-section and single-slide cohorts. P values were calculated using Wilcoxon rank-sum test. The number of samples in each dataset is indicated in the figure. **F)** Number of proteins (gene-centric, left) and peptides (right) identified per sample in the single-slide cohort timsTOF dataset, in relation to the injected sample amount. **G)** Number of proteins (gene-centric) identified per sample in the single-slide cohort in relation to the total MS signal expressed as the sum of precursor MS1 signal, shown for the the Astral (left) and timsTOF (right) datasets.

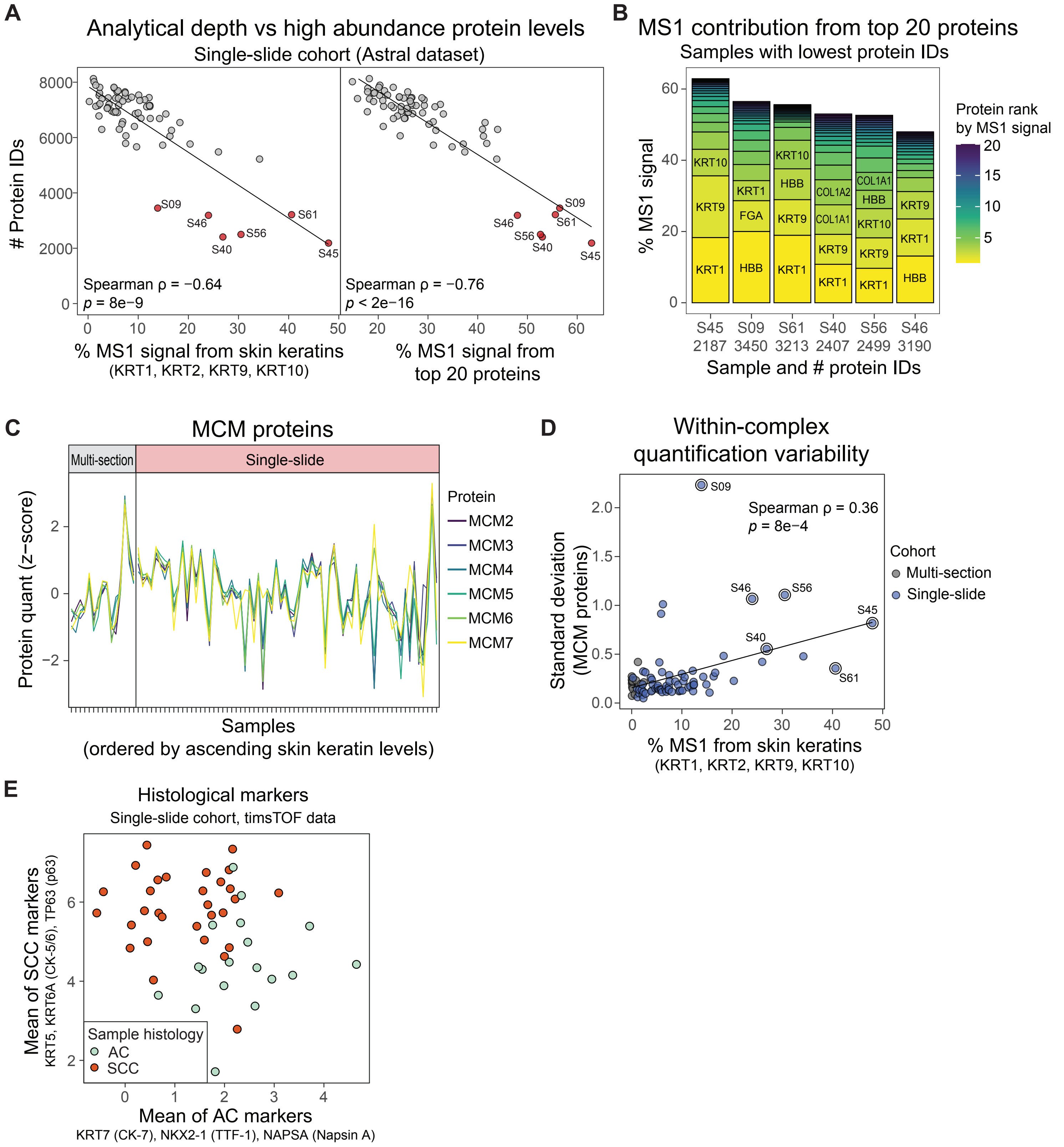

Supplementary Figure 2. The effects of high abundance proteins on analytical depth and quantification; and histological marker evaluation (related to Figures 1 and 2). A) Number of proteins (gene-centric) identified per sample in the single-slide cohort Astral dataset in relation to the percentage of MS1 signal attributed to high-abundance proteins: four skin keratins (left) and top 20 proteins by signal (right). **B)** Percentage of MS1 signal from the 20 most abundant proteins in the six samples that had < 5000 identified proteins. Proteins contributing >5% of MS1 signal are annotated. **C)** MCM complex protein quantifications, expressed as z scores derived from scaling across the cohort. **D)** The standard deviation of MCM protein quantification as shown in panel C in relation to the percentage of MS1 signal attributed to the four skin keratins. **E)** Histological markers in adenocarcinoma (AC) and squamous cell carcinoma (SCC) samples (n = 48) in the single-slide cohort timsTOF dataset.
